# Endoscopic hot snare resection of a type III biliary cyst (choledochocele)

**DOI:** 10.1055/a-2446-2072

**Published:** 2024-11-08

**Authors:** Andrea Sorge, Stefano Mazza, Francesca Torello Viera, Aurelio Mauro, Davide Scalvini, Alessandro Vanoli, Andrea Anderloni

**Affiliations:** 1 9304 Pathophysiology and Transplantation, University of Milan, Milan, Italy; 2 60200 Gastroenterology and Hepatology, University Hospital Ghent, Ghent, Belgium; 3 18631 Gastroenterology and Endoscopy Unit, Fondazione IRCCS Policlinico San Matteo, Pavia, Italy; 4 18631 Unit of Anatomic Pathology, Fondazione IRCCS Policlinico San Matteo, Pavia, Italy; 5 607734 Unit of Anatomic Pathology, University of Pavia Department of Molecular Medicine, Pavia, Italy

**Fig. 1:**
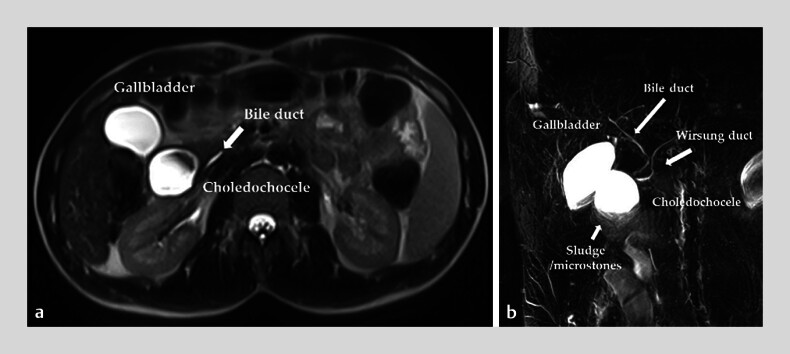
Magnetic resonance cholangiopancreatography images showing a cystic formation close to the duodenal wall, with hypodense material in the dependent portion, consistent with biliary sludge/stones, and without clear communication with the bile duct, along with normal-appearing bile and Wirsung ducts on: **a** transverse view; **b** coronal view.

A 24-year-old man was referred to our unit for an incidentally diagnosed duodenal cystic lesion. Magnetic resonance cholangiopancreatography revealed a cystic lesion, measuring 30 × 35 mm, located near the major papilla, protruding into the duodenal lumen ( Fig. 1 ) and containing biliary stones.

**Fig. 2:**
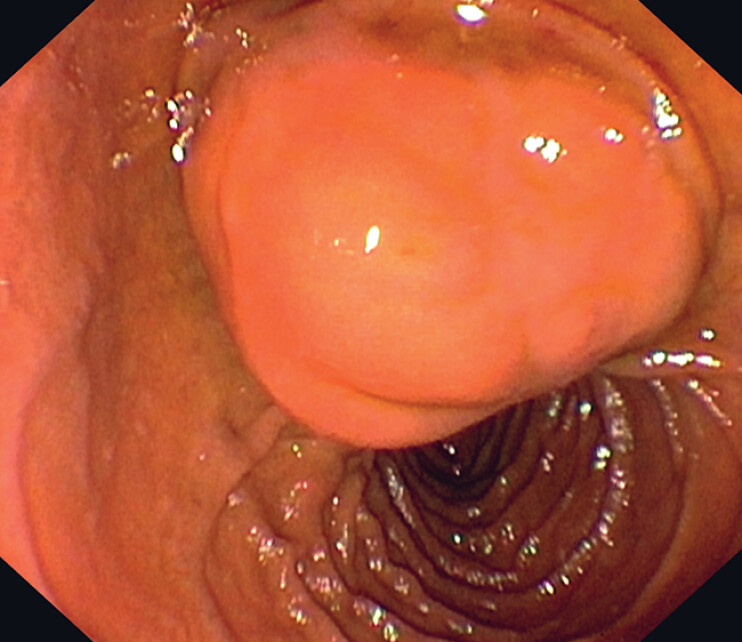
Endoscopic image of the type III biliary cyst (choledochocele), which appeared as a roundish lesion surrounded by normal duodenal mucosa.

**Fig. 3:**
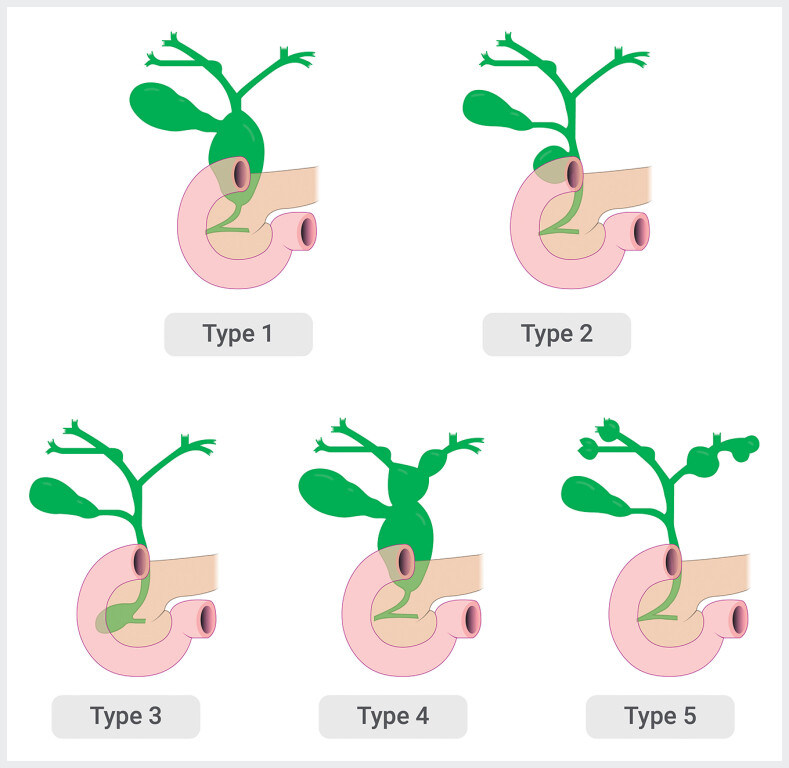
Schema of the Todani classification of biliary cysts.

A side-viewing duodenoscopy revealed a roundish subepithelial lesion in the descending duodenum, which was covered by normal-looking mucosa ( Fig. 2 ). The papillary orifice was located in the distal portion of the lesion. An endoscopic ultrasound confirmed an anechoic cystic lesion of 44 × 38 × 35 mm containing biliary stones. The common bile duct diameter and opening into the duodenum were normal. A diagnosis of a Todani type III biliary cyst (choledochocele) was made ( Fig. 3 ).

After multidisciplinary discussion, the patient was referred for endoscopic resection. With the patient under deep sedation with propofol, the choledochocele was resected en bloc using the hot snare papillectomy technique using a side-viewing duodenoscope ( Video 1 ). A 20-mm snare with a blended cut–coagulation current (Endocut Q effect 2; ERBE Vio3, Tübingen, Germany) was used. After the resection, clear bile and biliary stones spontaneously drained from the bile duct. The bile duct proximal to the resection site appeared uninjured, so we decided not to place clips on the mucosal defect. To prevent post-papillectomy pancreatitis, an attempt was made to place a pancreatic stent; however, this could not be placed owing to difficult pancreatic duct cannulation.

A type III biliary cyst (choledochocele) is successfully resected en bloc with hot snare resection.Video 1

No adverse events occurred during the procedure and the post-procedural course was uneventful. The patient was discharged the day after the procedure. Histologic examination confirmed a biliary cyst with both the internal and external surfaces lined by nondysplastic duodenal mucosa. At the 3-month follow-up, the patient was asymptomatic and duodenoscopy revealed normal bile flow with no residual lesions.

Endoscopic resection is a safe and effective treatment strategy for Todani type III biliary cysts (choledochoceles).

Endoscopy_UCTN_Code_CCL_1AZ_2AK

